# One-Step Synthesis of Long Term Stable Superparamagnetic Colloid of Zinc Ferrite Nanorods in Water

**DOI:** 10.3390/ma12071048

**Published:** 2019-03-29

**Authors:** Angelika Kmita, Dorota Lachowicz, Jan Żukrowski, Marta Gajewska, Wojciech Szczerba, Juliusz Kuciakowski, Szczepan Zapotoczny, Marcin Sikora

**Affiliations:** 1 Academic Centre for Materials and Nanotechnology, AGH University of Science and Technology, al. A. Mickiewicza 30, 30-059 Krakow, Poland; dbielska@agh.edu.pl (D.L.); zukrow@agh.edu.pl (J.Ż); marta.gajewska@agh.edu.pl (M.G.); w.szczerba@post.pl (W.S.); jkuciako@agh.edu.pl (J.K.); marcin.sikora@agh.edu.pl (M.S.); 2 Faculty of Physics and Applied Computer Science, AGH University of Science and Technology, al. A. Mickiewicza 30, 30-059 Krakow, Poland; 3 Faculty of Chemistry, Jagiellonian University, Gronostajowa 2, 30-387 Krakow, Poland; zapotocz@chemia.uj.edu.pl

**Keywords:** zinc ferrite, nanorods, colloids, magnetic properties, functional materials

## Abstract

Synthesis of spinel zinc ferrite ultrafine needle-like particles that exhibit exceptional stability in aqueous dispersion (without any surfactants) and superparamagnetic response is reported. Comprehensive structural and magnetic characterization of the particles is performed using X-ray and electron diffraction, small angle X-ray scattering, transmission electron microscopy, dynamic light scattering, vibrating sample magnetometry, Mössbauer spectroscopy and high-resolution X-ray spectroscopy. It reveals nearly stoichiometric ZnFe_2_O_4_ nanorods with mixed spinel structure and unimodal size distribution of mean length of 20 nm and diameter of 5 nm. Measurements performed in aqueous and dried form shows that particles’ properties are significantly changed as a result of drying.

## 1. Introduction

### 1.1. Ferrite Nanoparticles Applications 

Ferrite nanoparticles with controllable morphology and crystal structure are attracting a significant amount of interest due to their extensive applications, ranging from fundamental research to industrial use [1]. The particle size of spinel ferrites is dependent on the nature of transition metal and the synthesis method. Forms of nanoparticles like spherical, nanorods, nanoflowers or nanotubes and their specific surface area play an important role in tuning their functional properties for applications [2,3,4,5].

One dimensional (1D) nanostructures e.g., nanowires, nanobelts, nanotubes and nanorods have been widely investigated due to their unusual electrical and optical properties, magnetism and mechanical properties that make them suitable for application in sensors, catalysis, drug delivery, hyperthermia, medical diagnostics or photoelectrochemical water splitting [6,7,8]. In most of these applications, the higher the active surface to volume ratio, the better efficiency of the process, provided that the material is well dispersed in the carrying medium.

Zinc ferrite (ZnFe_2_O_4_) nanoparticles, the subject of this research, are often used as catalysts, for instance in methanol decomposition into CO and hydrogen, decomposition of greenhouse gases (CO_2_), or in oxidative organic reactions [6,9,10]. They have been also used in the foundry industry, sensors technology and lithium ion batteries [11,12]. Moreover, nanoparticles of zinc ferrite were used as functional materials for applications in casting technologies or as heating agents for in vitro hyperthermia assay on glioma cells [13,14].

### 1.2. Synthesis Methods

1D zinc ferrite nanoparticles can be synthesized by numerous methods. For example, Liu et al. prepared ZnFe_2_O_4_ nanotube arrays using porous anodic aluminium oxide template from sol-gel solution [15], while Li et al. obtained ZnFe_2_O_4_ nanofibers by thermal treatment of the fibre-like precursor [16]. J. Zhao et al. presented a simple strategy for preparation of zinc ferrite nanorods by decomposition of ferrocenyl complex Zn(fca)_2_ without the assistance of catalysts or template [17]. Porous zinc ferrite nanorods with subsequently light-driven photocatalytic activity were synthesized by the thermal decomposition of ZnFe_2_(C_2_O_4_) [6]. Prepared in solution and deposited on F-doped tin oxide glass substrate, 1D zinc ferrite nanorods form a high efficiency photoanode for water splitting [7]. Finally, zinc ferrite nanorods films for sensing applications were successfully prepared by employing sol-gel spin coating process using zinc sulphate and ferric nitrate [2].

### 1.3. Applications of Colloidal Dispersions

Colloidal dispersions of ferrite nanoparticles are intensively investigated in order to test their applicability in medicine: in cancer therapy using magnetic hyperthermia, as MRI contrast agents, for DNA hybridization, drug delivery; as well as in the industry, as magnetic materials, used for example in linear and rotary dampers (shock absorbers, clutches, brakes), in step motors (damping of vibrations), in seals (in vacuum technique) [18,19,20,21,22] etc.

### 1.4. Stability of Colloidal Dispersion

The water colloidal stability of nanostructures of various shapes and sizes constitutes the most important challenge to achieve useful systems for biomedical and industry applications. Preparation of colloidal stable dispersion is usually performed in two stages:

(1) Formation of nanoparticles, e.g., zinc ferrite ZnFe_2_O_4_, by means of e.g., micro-emulsion [23], co-precipitation method [24,25], milling [26], sol-gel method [27], hydrothermal [28], solvothermal [29], spray pyrolysis [30] or thermal decomposition method [31],
(2) Further stabilisation/dispersion of nanoparticles in various non-polar or polar fluids [32,33].

There are two modes of stability of colloidal dispersion. The first is the kinetic stability of the system (relative to gravity forces). The crucial factors that determine the kinetic stability of colloids are mainly Brownian motion and viscosity of the medium. The second mode is aggregation stability of the system. In this case nanoparticles can create large aggregates absorbing on their surface low-molecular ions from the solution, leading to the formation of an adsorption layer [34].

Many different strategies to transfer the as-synthesized nanocrystals into aqueous media are presented in the literature. One can use either inorganic (e.g., silica, gold) or polymeric coatings, e.g., chitosan, glucose, dextran, poly(ethylene glycol) (PEG), poly(vinylalcohol) (PVA), in order to increase the water colloidal stability [35,36,37], or surfactants such as sodium oleate, dodecylamine, dopamine, silane agents or acid groups, in order to coordinate metal ions at the nanostructure surface (steric repulsion) and to prevent aggregation [1,33,38]. The nanoparticles can also be prohibited from sticking to each other by electrostatic repulsive charged coating layer, which leads to particles stability in polar solvents like e.g., aqueous media [33]. Strength of electrostatic and/or steric repulsion are the key parameters to elaborate particles solutions with good stability. However, steric forces are difficult to predict and quantify, and while the electrostatic repulsion can be ascertained through knowledge of the zeta potential, and the Debye-Huckel length, which depends on the pH of the solution and on the ionic strength [34,39], nevertheless, it is still an open challenge to develop synthesis procedures resulting in stable colloidal dispersions of ferrite nanoparticles on a large scale and at low cost.

### 1.5. Present Research

In this paper we report on the properties of the stable colloid formed by nanorods of zinc ferrite diluted in water, obtained as a result of single stage green reaction, namely co-precipitation from aqueous salt solution in an alkaline medium.

## 2. Materials and Methods 

### 2.1. Materials

Materials used in this study: iron (III) chloride hexahydrate (98%); zinc (II) chloride (≥98%) from Sigma–Aldrich and NaOH (100%) from POCH. Solutions were prepared using ultra-pure water (18.2 MΩ cm at room temperature) as the solvent.

#### Synthesis of ZnFe_2_O_4_ Nanorods in Water Solution

**Scheme 1:**
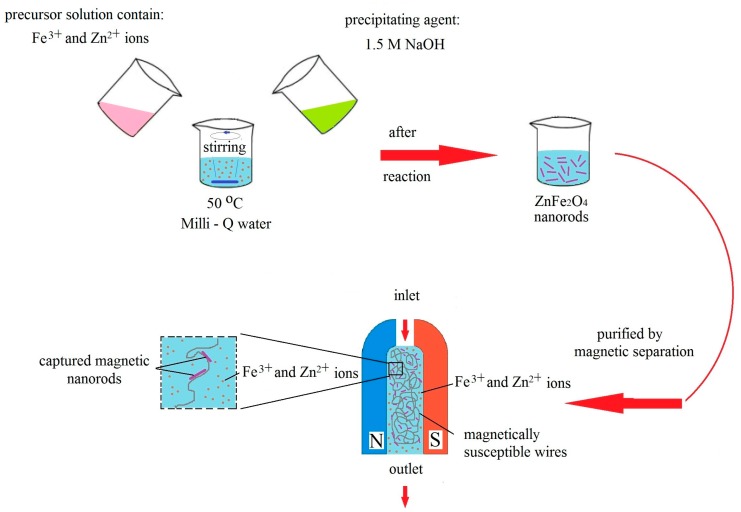
Procedure followed to obtain ZnFe_2_O_4_ nanorods in water solution.

ZnFe_2_O_4_ nanoparticles were prepared by co-precipitation method, [24,25] which was modified as shown in the Scheme 1. Iron (III) chloride hexahydrate (Sigma-Aldrich, St. Louis, MO, USA), zinc (II) chloride (Sigma-Aldrich), and sodium hydroxide NaOH (POCH, Gliwice, Poland) were used. The precursor solution was prepared by dissolving ZnCl_2_ (16.5 mmol) and FeCl_3_·6H_2_O (33.5 mmol) in water. Composition of the solution of metal salts was selected to contain Fe^3+^ and Zn^2+^ ions in the molar ratio of 2:1. As the precipitating agent water solution of NaOH (1.5 M) was used (25 mL).

Sodium hydroxide and the precursor solution were added dropwise to the demineralized water heated to 50 °C. The reaction vessel was heated for 6 hours under magnetic stirring. Afterwards, the obtained dispersion of nanoparticles was cooled and purified by magnetic separation. This process was based on passing the crude dispersion through a column filled with steel wool in the presence of the permanent magnetic field. The column was washed out by water and the stable colloid with nanorods of zinc ferrite was obtained. The process was repeated several times in a reproducible manner. Further research was performed on (1) the dispersion of the nanorods with the total concentration of iron equal to c = 0.56 mg·cm^−3^ as well as on (2) dry particles obtained by water evaporation. 

### 2.2. Methods

#### 2.2.1. X-ray Powder Diffraction (XRD)

The crystalline structure and composition of the zinc ferrite nanorods were analyzed by X-ray powder diffraction (XRD). Diffraction patterns were recorded for the dried sample and compared to the reference ZnFe_2_O_4_ sample (ICSD: 98-009-1932). Measurements at room temperature, were performed using Panalytical Empyrean diffractometer (Royston, UK) using Cu Kα radiation (1.541874 Ǻ) at 40 kV and 40 mA. The data were collected in the Bragg–Brentano geometry using 0.026 step at 3000 s/step. 

#### 2.2.2. Transmission Electron Microscopy (TEM) 

The morphology and microstructure of the particles were studied using TEM. Micrographs were obtained at 200 kV from FEI TECNAI TF20 X-TWIN (Hillsboro, OR, USA) high resolution microscope equipped with field emission gun, with a point resolution better than 0.25 nm. Selected area electron diffraction (SAED) patterns were also acquired. TEM sample preparation was done by putting a droplet of the colloidal solution of ZnFe_2_O_4_ nanorods in water on a holey TEM Co grid coated with carbon film. Then the sample was dried in air at room temperature.

#### 2.2.3. Small Angle X-ray Scattering (SAXS)

The shape and size of the particles was determined in water solution by SAXS. The scattering curves were recorded using a slit focus Kratky system by Anton Paar (Gratz, Austria), equipped with a Mythen 1D CMOS array detector by Dectris (Baden, Switzerland). The sample holder was a SAXS proof plastic tube capillary. 

#### 2.2.4. Mössbauer Spectroscopy

Phase composition of dried particles was probed using ^57^Fe Mössbauer spectroscopy. Measurements were performed using Renon MS-4 spectrometer (Krakow, Poland) at 80 K and 300 K, with the thermal stability of the order of 0.05 K, on the micrometer thick film of particles prepared by means of dropcasting. 

#### 2.2.5. Near Edge X-ray Absorption Structure (XANES) 

The atomic structure of particles in solution was also determined using iron K-edge XANES spectroscopy accomplished at ID26 beamline at ESRF, Grenoble, France. Double crystal Si(311) incident beam monochromator and Ge(440) emission analyzers were used for the measurements of Kα high energy resolution fluorescence detected (HERFD) spectra. Such an experimental scheme allowed for probing spectra of dried particles as well as in water solution. The latter was possible due to the efficient filtering of high intensity background counts of the scattering on solvent [40,41].

#### 2.2.6. Dynamic Light Scattering (DLS) and Zeta Potential Measurement

For the measurement of particle size (by DLS method) and Zeta potential (LDV), a Malvern Nano ZS instrument (Malvern Instrument Ltd., Worcestershire, UK) was used.

All samples were illuminated using a 633 nm laser (constant angle of 173°). The detector was a photodiode. The Z-average hydrodynamic diameter (D*_h_*) values and dispersity index were (PdI) calculated using the ZetaSizer software (version 7.12, Malvern).

#### 2.2.7. Vibrating Sample Magnetometry (VSM)

Magnetization measurements were performed in the temperature range of 80–300 K using LakeShore 7407 VSM (Westerville, OH, USA) magnetometer equipped with liquid nitrogen continuous flow cryostat made by Janis Research Company (Carson, CA, USA).

#### 2.2.8. Magnetic Hyperthermia

Water solution of ZnFe_2_O_4_ nanorods (*c*_Fe3+_ = 0.56 mg cm^−3^) was investigated using the induction heating system based on AMBRELL EasyHeat generator (St. Louis, MO, USA). The water-cooled coil consisting of 7 turns, 2-inch-long and 1-inch internal diameter, was powered by AC current up to 250 A at a frequency equal of 360 kHz. 

## 3. Results and Discussion

### 3.1. X-ray Powder Diffraction and Transmission Electron Microscopy

**Figure 1:**
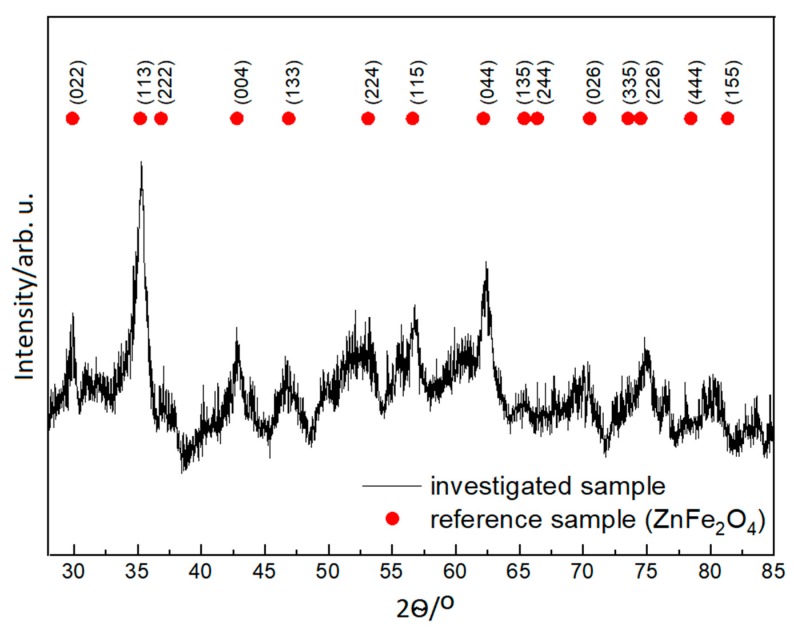
XRD pattern of nanorods of ZnFe_2_O_4_ (for dried nanoparticles after synthesis by co-precipitation method).

In Figure 1 the XRD pattern of the dried nanorods is compared to that of the reference ZnFe_2_O_4_ sample. It reveals that the ordered volume of the dried particles consists predominantly of nano-crystalline spinel. The pattern can be indexed as zinc ferrite. The most pronounced peaks at increasing 2θ correspond to 022, 113, 004 and 044 crystal planes of spinel ZnFe_2_O_4_ (ICSD: 98-009-1932), respectively. The mean particle size estimated using the Scherrer equation applied to the analysis of the highest intensity diffraction peak, namely the (113) reflection, was 14 nm. A significant broadening of the diffraction peaks visible in Figure 1 is characteristic for the diffraction of small particles (nanoparticles) [12]. Diffraction pattern obtained is in line with that reported by Z. Jia et al. for 100–200 nm long porous zinc ferrite nanorods prepared by thermal decomposition method [6]. 

The pattern shown in Figure 1 is similar to that obtained by A. Singh et al. from the nanorods of pure cubic phase of ZnFe_2_O_4_ with minimum crystallite size of 10 nm [2]. However, the stronger broadening of XRD peaks in our case suggests that the investigated sample consists of significantly smaller crystallites, as confirmed by TEM.

### 3.2. TEM Images and Selected-Area Electron Diffraction (SAED) 

**Figure 2:**
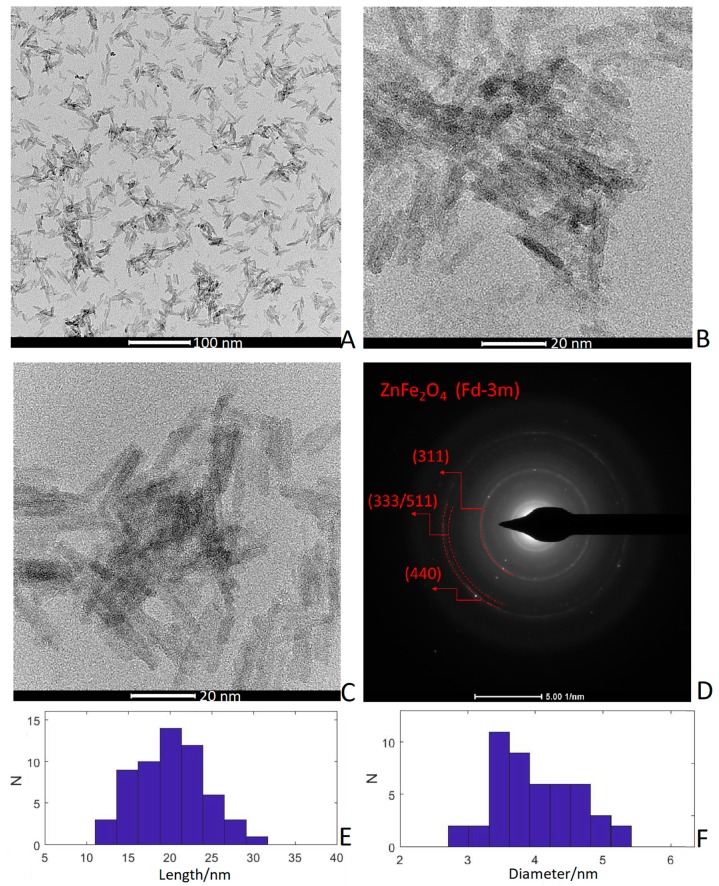
(**A**–**C**) TEM images of zinc ferrite nanorods, (**D**) their selected-area electron diffraction (SAED) pattern, (**E**,**F**) histograms respectively for length: mean = 19.9 nm, std/mean = 22% and for width mean = 4.02 nm, std/mean = 16%.

Micrographs with size distribution histograms and SAED patterns of zinc ferrite nanoparticles are presented in Figure 2. It is clear that the nanoparticles of ZnFe_2_O_4_ are nearly monodisperse in size and shape. They have the form of rods with a length of approximately 10–20 nm and a thickness of around 3–5 nm. Furthermore, the rings of SAED patterns are related to the diffraction of the zinc ferrite with nanocrystalline structure, which is consistent with the XRD results.

### 3.3. Small Angle X-ray Scattering

**Figure 3:**
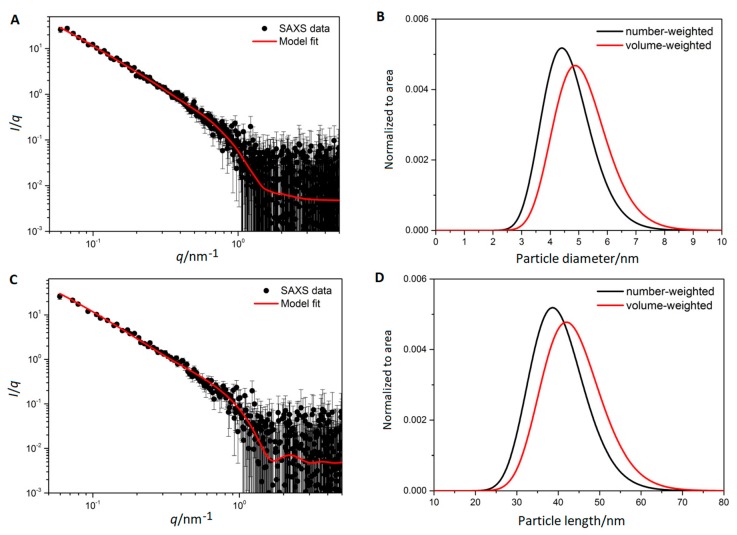
Scattering curve of zinc ferrite nanorods in aqueous dispersion and its fit modelled with cylindrical particles of unique length (**A**) and log-normal distribution of rod diameters (**B**). Below are: fit modelled with cylindrical particles of unique radii (**C**) and log-normal distribution of rod lengths (**D**).

The Guinier plots, resulting from SAXS data reduction, are shown in Figure 3. The evaluation of the data was performed using two independent fits. In the first fit the scattering curve was modelled by distribution of cylindrical particles with a log-normal distribution superimposed on their radii, while the length of the particles was constrained to single value [42,43]. The fit results indicated that aggregates were present in the sample. They were modelled by a mass fractal with exponential cut-off [44]. A second fit was done assuming log-normal distribution superimposed on the particle length with a fixed value of the radius. The latter was equal to the mean value obtained from the first fit. Also, the structure factor was unchanged. A presented fit sequence was chosen because SAXS is more sensitive to the diameter of rod-like objects [44]. The results of both fits indicated that the particles were primarily rods with a moderately broad diameter distribution. The mean diameter was 4.64 nm (Figure 3B) and the mean length of the rods was 40.2 nm (Figure 3D) with a standard deviation of 0.84 nm and 6.6 nm, respectively. A rod-like shape of the particles and the structure of aggregates probed from large volume of the dispersion investigated is consistent with observations made by electron microscopy.

### 3.4. Zeta Potential (ζ) and Dynamic Light Scattering

The determined electrokinetic potential (ζ) of zinc ferrite nanorods in the water solution oscillated within +47.7 ± 2.4 mV (Table 1). For various colloidal systems there is a pH value, at which the electrokinetic potential equals zero (isoelectric point) and the electrostatic repulsion does not counteract colloid coagulation [1,3]. As it is well known, the value of app. +/–30 mV is assumed in water systems as the boundary value determining the dispersion stability [3]. In the analysed case, the high value of Zeta (ζ) potential (+50 mV) assures the stability of the dispersion of nanorods by electrostatic repulsion. No sedimentation was observed in bottles of nanorods, even after 4 months of storage at 4 °C at pH = 7.0, indicating that the dispersion of zinc ferrite nanorods in water is extraordinarily stable. 

**Figure 4:**
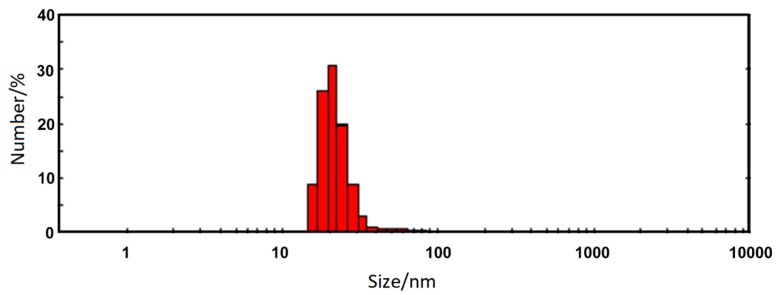
Distribution profiles of the hydrodynamic diameters (*D*_h_) obtained from DLS measurements for the nanorods.

Hydrodynamic size (*D*_h_) was measured using dynamic light scattering (Figure 4). The measurements showed that ZnFe_2_O_4_ nanorods in the aqueous dispersion were in the form of isolated nanorods dominate in the aquesous dispersion as the measured hydrodynamic diameter reflect the largest dimension of the nanorods. The average hydrodynamic diameter (*D*_h_) is 22.5 ± 7.9 and the dispersity index (PdI) was found to be equal 0.26 (Table 1).

### 3.5. Mössbauer Spectroscopy

**Figure 5:**
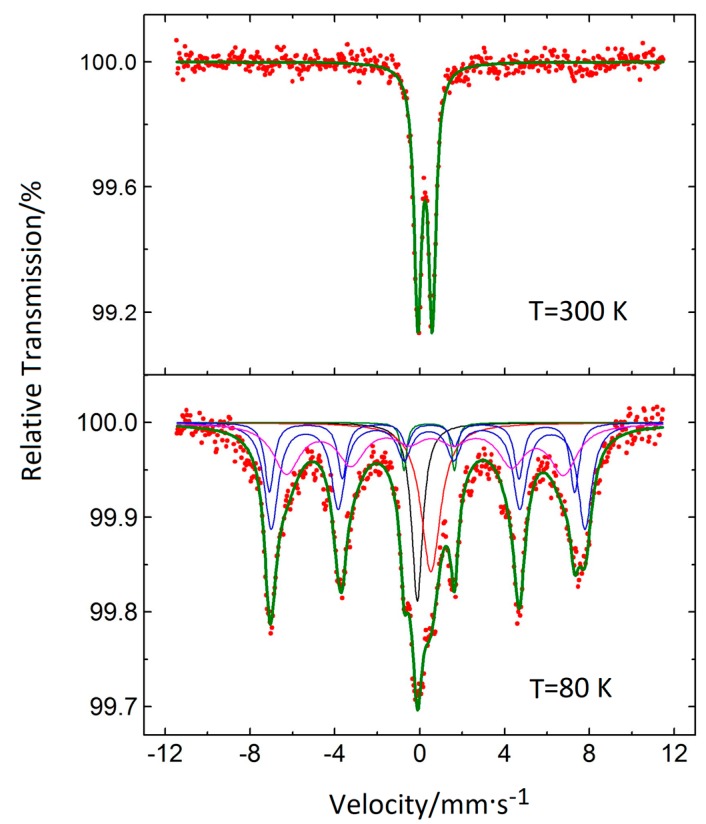
57Fe Mössbauer spectra measured at 300 K and 80 K for nanorods of zinc ferrite (red dots) along with the result of multicomponent fit (green solid line). Thin solid lines represent the spectra of individual components of spectrum collected at 80 K.

The structural characterization and phase composition of the nanorods were investigated using local spectroscopic probes involving nuclear and electronic interactions. Mössbauer spectra were measured for dry nanorods of zinc ferrite at room (300 K) and cryogenic (80 K) temperature (Figure 5). The spectrum probed at room temperature reveals a narrow line doublet. It was a typical relaxation spectrum observed for superparamagnetic iron oxide nanoparticles well above blocking temperature [45]. In line with TEM micrographs, it confirmed that the sample investigated was composed predominantly of nanoparticles of the mean volume significantly smaller than 500 nm^3^. Lowering the temperature of the measurement reduced the influence of thermal relaxation and better adjusted the hyperfine interaction parameters allowing for the chemical and structural phase analysis. In comparison to the other studies performed on spherical zinc ferrite nanoparticles of similar volume [45,46] we observed that the thermal relaxation effect at cryogenic temperature was significantly less affecting the linewidth of the spectral features in the case of nanorods. It was most likely related to the stabilizing effect of increased magnetic anisotropy due to strongly elongated shape of the particles. 

Mössbauer spectrum measured at *T* = 80 K shows several clearly separated nonmagnetic and magnetically split components—singlets, quadrupole doublets and magnetic sextets. A detailed numerical analysis of the spectrum was performed by fitting the experimental data with six independent contributions, each assuming a distribution of hyperfine parameters resulting from the particle size distribution. Table 2 summarizes the fit parameters and provides the best fit values of the individual components, namely isomer shift (IS), magnetic hyperfine field (H) and quadrupole splitting (QS). A fit consisting of at least six spectral contributions was necessary to obtain good agreement to all the features visible in the spectrum. These components are three sextets, one doublet and two singlets. Singlets and doublets are associated with the paramagnetic iron ions at the particle surface. Their high fraction is in line with large surface to volume ratio due to small diameter and elongated shape of the particles studied. The observed number of disordered sites, nearly 30% of all Fe sites, corresponded to a surface shell that was approximately 0.5 nm thick. Three magnetically split components attributed to iron sites within the core of the particle, namely components 4–6, revealed a mean isomer shift of <IS> ≈ 0.39 mm·s^−1^ and small quadrupole splitting indicating that the ordered inner volume consists mainly of Fe^3+^ ions. Mean value of magnetic hyperfine field of these components, <H> = 435.3 kGs, was consistent with that observed in spherical zinc ferrite nanoparticles [47,48].

Provided that the <IS> and <H> dependence on the Zn content shown in Reference [47] can be extrapolated to higher Zn doping, the parameters observed for nanorods studied correspond to a nearly stoichiometric ZnFe_2_O_4_ core. However, stoichiometric spinel ferrite should reveal only two magnetically split components, corresponding nominally to Fe^3+^ in tetrahedral and octahedral local environments, and a hyperfine field reduced due to non-magnetic ions in the second coordination shell. Upon comparison of these parameters with the results obtained for magnetite in the form of single crystals [49] and nanoparticles [50] we attribute component four to the tetrahedral site and component six to octahedral spinel site. Their relative contributions are in line with inverse spinel structure of nearly stoichiometric zinc ferrite. The remaining contribution (component five) is characterized by significantly lower isomer shift. It is associated to octahedral iron sites in magnetic phases other than spinel and tentatively ascribed to a possible hematite contribution at particles’ surface. We address this issue in the discussion of X-ray absorption spectroscopy results (Section 3.6).

### 3.6. X-ray Absorption Spectroscopy

**Figure 6:**
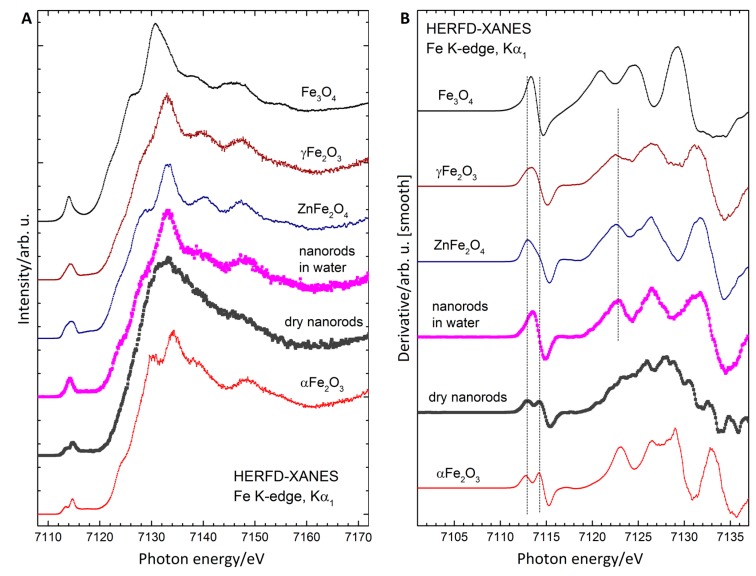
Fe K-edge HERFD-XANES spectra of zinc ferrite nanorods compared to reference spectra of selected iron oxides (**A**) and first derivative of the smoothed HERFD-XANES (**B**).

Further insight into the local atomic environment of Fe ions, and thus the dominating structural phase in the composition of the nanorods, is achieved by means of X-ray absorption near edge structure. Although the spectral features of XANES are less pronounced than that of Mössbauer spectra they are well suited for probing nanoscale materials due to the significantly shorter characteristic time of core-level electronic transitions involved [51]. Normalized iron K-edge HERFD-XANES spectra of nanorods are compared with that of reference iron oxides on Figure 6. The pre-edge, which is visible in the incident photon energy range of 7112–7118 eV, is a fingerprint of local crystal symmetry and electronic occupation of iron sites. In the case of only octahedrally coordinated Fe^3+^, as in the case of hematite (α-Fe_2_O_3_), the pre-edge consists of two separated peaks of relatively low intensity. The pre-edge of tetrahedral sites is characterized by a strong single feature that dominates the average spectral shape in the case of magnetite and maghemite spectra [52]. 

The main edge region, range beyond 7118 eV, is shaped by next neighbors (long range crystal structure) and shifted to higher energy with increasing mean oxidation state of iron ions [53]. The characteristic step-like shape of the rising edge due to the superposition of the signal from crystal sites of different symmetry [54], as well as edge shift, can be well discerned from the smoothed derivative spectra shown in Figure 6B. 

HERFD-XANES spectra of zinc ferrite particles were collected from both stable water dispersion and from dry nanorods. Their comparison indicates a striking difference. Upon drying the pre-edge range evolves from single to double-peak while the edge becomes much less structured. The latter is clearly manifested in the transformation of the derivative spectrum from multi-peak structure to broad distribution characteristic to amorphous or strongly disordered, multiphase systems. The spectrum obtained from the solution of nanorods resembles well that of ZnFe_2_O_4_ reference nanopowder in the edge region. The characteristic three maxima visible in the derivative spectrum in the energy range of 7120–7133 eV reveal a nearly identical energy position and similar intensity. It is a clear indication of the dominating volume of spinel phase in the atomic structure of the nanorods studied [41]. Energy of the first maximum, i.e., 7122.8 eV, is characteristic to that observed in maghemite (γ-Fe_2_O_3_) and hematite references indicating that nominally Fe^3+^ iron ions are present only. However, a clear discrepancy between the spectra of nanorods in solution and ZnFe_2_O_4_ reference is observed in the pre-edge region. Main peak intensity is significantly stronger in the nanorods spectra that clearly demonstrates a significant amount of tetrahedral Fe sites, i.e., spinel inversion. In the case of dry nanorods spectrum, a similar edge position as that of other nominally Fe^3+^ oxides spectra is revealed, but the edge shape is significantly less structured, manifested by single broad maximum in the first derivative. It is attributed to a significant structural disorder, which is in line with Mössbauer data fit suggesting that over 40% of iron atoms present in the sample are not forming spinel phase. On the other hand, the spectral shape of the pre-edge is better resolved. It resembles well that of hematite, which is predominantly formed by nominally Fe^3+^ ions in octahedral crystal field, although some presence of tetrahedral sites is clearly manifested by smaller depression between the two maxima in the derivative spectrum. As such we postulate that disordered non-magnetic phases and hematite phase volumes observed in Mössbauer spectra are characteristic to drying process, while they are negligible in the nanorods kept in water.

### 3.7. Magnetic Properties

Based on the findings of structural characterization described earlier, namely that the nanorods in dispersion are single phase strongly anisotropic particles formed predominantly from nearly stoichiometric ZnFe_2_O_4_ in mixed spinel structure, it is expected that they manifest weak ferromagnetic or superparamagnetic properties. The drying process, which transforms large fraction of particle volume into low symmetry non-magnetic oxide phases, shall affect their magnetic properties significantly. These expectations are indeed confirmed by DC magnetometry. 

**Figure 7:**
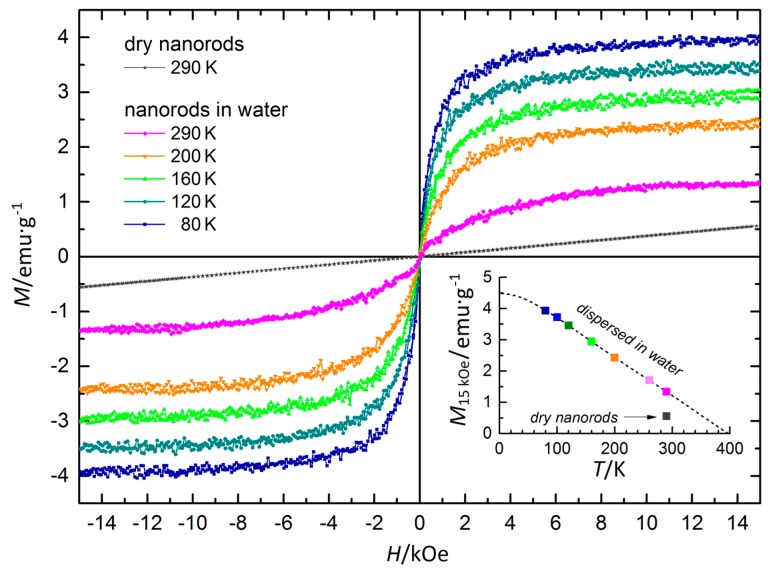
Magnetization curves of ZnFe_2_O_4_ nanorods dispersed in water upon subtraction of diamagnetic contribution from solvent and container. Inset shows the dependence of high field magnetization versus temperature (line is a guide for the eye).

Figure 7 shows the magnetization (*M*(*H*)) loops of zinc ferrite nanorods. The *M*(*H*) profiles of the particles in water exhibit a clear tendency to saturation in moderate magnetic fields, revealing weak superparamagnetic behavior in the whole temperature range probed. On the other hand, the magnetization profiles of dry particles exhibit a linear dependence characteristic for antiferromagnetic and/or paramagnetic materials. The lack of magnetic hysteresis implies that magnetic blocking temperature of nanorods in water solution is lower than 80 K. It is not surprising provided their tiny dimensions and small value of saturation magnetization is estimated at 4–5 emu·g^−1^. 

The *M*(*T*) profile extracted from magnetization profiles at *H* = 15 kOe shows a typical linear dependence characteristic for ferrimagnetic oxides. In the case of ZnFe_2_O_4_ the finite magnetization observed can be attributed to the mixed spinel structure or cationic nonstoichiometry. Provided the unlikely case that only the latter scenario is realized, the amount ratio of Fe:Zn differs from the nominal 2:1 value by less than 5%. Matching the *M*(*T*) dependence by the curve typical for spinel ferrites extrapolates the Curie temperature of the nanorods in water solution to be close to 400 K, which confirms a strong interaction between iron ions building the spinel structure.

**Figure 8:**
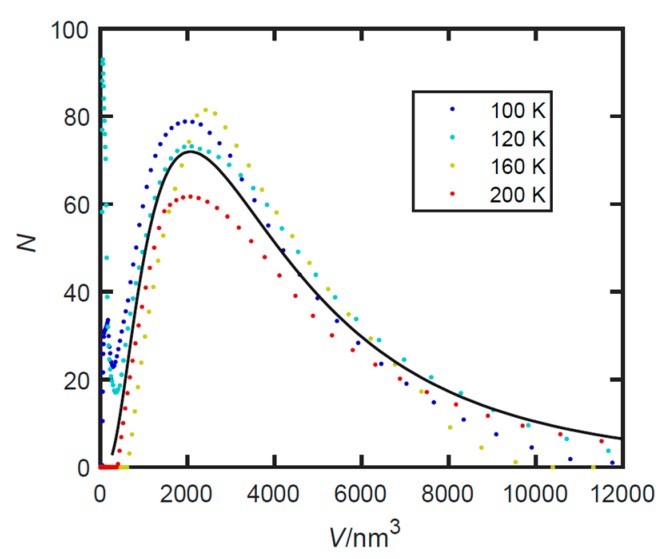
Particle volume distribution obtained using MINORIM software by inversion method analysis of magnetization loops. Profiles obtained from analysis of data collected in a wide temperature range (dots) were fitted with unique log-normal distribution function (solid line).

Hysteresis loops can be fitted using a set of Langevin functions. Each function corresponds to fraction of particles with defined magnetic moment *µ*_i_, and the whole set that best fits the curvature of measured loop corresponds to distribution of magnetic moments in the sample. Measured value of saturation magnetization can be used to recalculate value of particle magnetic moment to its volume using *V*_i_=*µ*_i_*/M* formula. Using MINORIM software [55] we have analysed magnetization profiles of the frozen solution. According to the analysis results (Figure 8), magnetic response corresponds to small agglomerates.

The mean is slightly less than 3000 nm^3^ that is about 3–5 nanorods per agglomerate on average. However, significantly larger magnetic agglomerates are also present, in line with TEM and DLS results. MINORIM results are well represented by log normal distribution around the peak, but there is significantly smaller fraction of large agglomerates than suggested by the tail of log normal distribution. Free particles not incorporated in the aggregates, i.e. smaller than 4 nm in diameter (approx. 300 nm^3^ in volume), can also be found.

### 3.8. Potential Application of ZnFe_2_O_4_ Nanorods Dispersed in Water in Magnetic Hyperthermia Therapy

The superparamagnetic behaviour and high temporal stability observed in the water solution of studied zinc ferrite nanorods are prerequisites for application in magnetic hyperthermia and related application. Hyperthermia as one of the forms of therapy supporting standard oncological therapy becomes very important in the field of medical physics, mainly due to the use of heating properties of magnetic nanoparticles, especially superparamagnetic particles of iron oxides. Heat induced by the AC magnetic field effect on superparamagnetic iron oxide nanoparticles, with a higher temperature in the range 41–45 °C, was used in hyperthermia therapy, especially for the treatment of breast cancer [56,57], for example.

In spite of decades of research, the hyperthermia effect in solution of magnetic nanoparticles is not fully understood [58]. There are several possible heating mechanisms proposed, which are associated with combination of hysteresis loss, viscous heating, eddy currents and susceptibility loss [59]. In superparamagnetic nanoparticles it is usually due to susceptibility loss, which reveals two relaxation times related with Brownian rotation of the nanoparticles in a liquid environment and the Neél rotation of particles’ magnetization direction [60,61].

The Specific Absorption Rate (SAR) and Cumulative Equivalent Minutes (CEM) rates are used in relation to the efficiency of hyperthermia [54]. SAR is also known as SLP (Specific Loss Power). It describes the amount of heat generated by nanoparticles at a given frequency. The higher the SAR W·g^-1^ value, the better. It is described by the Formula (1) [62]:

SAR= c (ΔT/Δt)(1)

where: c—thermal capacity of the material used, J·K^−1^; ΔT/Δt—is the temperature increase in the set time, °C·s^−1^.

**Figure 9:**
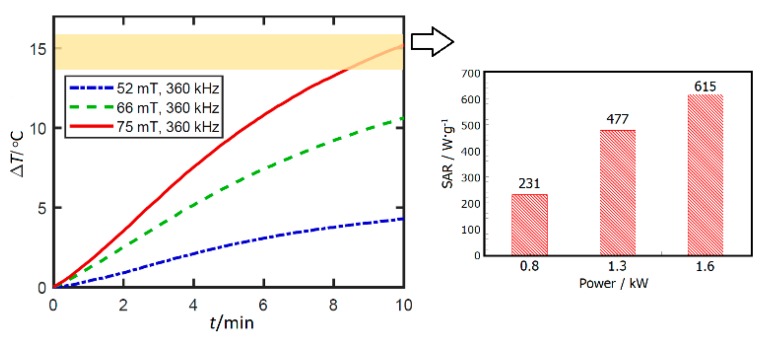
Temperature increase during 10 min of magnetic heating of ZnFe_2_O_4_ nanorods in water at iron ions (Fe^3+^) concentration of c = 0.56 mg·cm^−3^ and SAR value.

Low value of effective magnetization of the nanorods may result in the low effective energy conversion, or SAR value. In order to test whether therapeutic window can be achieved using the dispersion of ZnFe_2_O_4_ nanorods, the hyperthermia measurements were performed. Temperature change was probed as a function of time at different amplitude of AC magnetic field on a fresh solution of zinc ferrite nanorods in water with a concentration of 0.56 mg·cm^−3^. Figure 9 shows the results of the test for various magnetic field amplitudes with frequency equal to 360 kHz. The temperature increments (ΔT) for this system in water were 4.5, 10.5 and 15°C, respectively for 52, 66 and 75 mT B field amplitude (blue, green and red lines). The temperature rise was not abrupt, but the low limit of therapeutic window of 41 °C can be achieved in 8.3 min under B field amplitude equal to 75 mT. The calculated SAR value was respectively: 231, 477 and 615 W·g^−1^ (see in Figure 9). Provided that the higher amplitudes are applied in typical therapeutic conditions and that the density of particles can be increased due to their expected low toxicity, the therapeutic window could be achieved in significantly shorter time.

## 4. Conclusions

We present a method of obtaining the water solution of superparamagnetic nanoparticles of zinc ferrite that exhibit high temporal stability without any surfactants. It is an eco-friendly route of synthesis zinc ferrite nanorods in inorganic solvent, namely water, at low temperature of 50 °C. Comprehensive structural characterization of the particles reveal a strongly anisotropic shape of needle like nanorods of unimodal size distribution with a mean length of approx. 20 nm and 5 nm diameter. Nanorods in as-synthesized solution are formed from nearly stoichiometric ZnFe_2_O_4_ in mixed spinel structure. Upon drying, due to highly anisotropic shape and small radii, a significant volume of the rods transforms to lower symmetry oxide phases. In this way the functional properties of the particles can be tailored to application. 

Although the nanorods studied reveal rather weak superparamagnetic properties, in view of the extraordinary temporal stability of their water solution and lack of ferrous Fe^2+^ ions, they are suitable for biomedical applications. We have shown that for magnetic hyperthermia, but possible use in other applications, e.g., MRI thermometry [63], can be considered as well. A stable solution of magnetic nanoparticles (ferrofluid) of zinc ferrite that forms a shell consisting of hematite-like oxides upon drying may also be suitable for application in water splitting technology [2,3,4,5,6,7,8,9] as well as a functionalising agent in metallurgy and foundry industry [11,13].

## Figures and Tables

**Table 1:** The hydrodynamic diameters and zeta potentials of ZnFe_2_O_4_ nanorods.

Sample	d_max_/nm	PdI	ζ/mV
Nanorods ZnFe_2_O_4_	22.5 ± 7.9	0.26	+47.7 ± 2.4

**Table 2:** Mössbauer spectrum (80 K) components parameters; spectral weight of components, C, isomer shift, IS (relative to ^57^Co(Rh) source at 300 K), magnetic hyperfine field H, quadrupole splitting QS, and line width, Γ/2. Mean values of isomer shift, <IS>, and hyperfine field <H>, are provided for magnetic components (4–6) only.

Component No.	C %	IS/mm·s^−1^	<IS>/mm·s^−1^	H/kGs	<H>/kGs	QS/mm·s^−1^	*Γ*/2/mm·s^−1^
1	10.3	−0.1170	-	-	-	-	0.312
2	15.4	0.5256		-		-	0.558
3	2.8	0.4424		-		1.1894	0.157
4	30.6	0.415	0.390	458.3	435.3	−0.022	0.394
5	13.1	0.310		445.8		−0.202	0.256
6	27.8	0.399		404.9		−0.152	0.771
